# Decadal variability of tropical tropopause temperature and its relationship to the Pacific Decadal Oscillation

**DOI:** 10.1038/srep29537

**Published:** 2016-07-12

**Authors:** Wuke Wang, Katja Matthes, Nour-Eddine Omrani, Mojib Latif

**Affiliations:** 1 GEOMAR Helmholtz-Zentrum für Ozeanforschung Kiel, Kiel, Germany; 2 Christian-Albrechts Universität zu Kiel, Kiel, Germany; 3 Geophysical Institute, University of Bergen and Bjerknes Centre for Climate Research, Bergen, Norway

## Abstract

Tropopause temperatures (TPTs) control the amount of stratospheric water vapour, which influences chemistry, radiation and circulation in the stratosphere, and is also an important driver of surface climate. Decadal variability and long-term trends in tropical TPTs as well as stratospheric water vapour are largely unknown. Here, we present for the first time evidence, from reanalysis and state-of-the-art climate model simulations, of a link between decadal variability in tropical TPTs and the Pacific Decadal Oscillation (PDO). The negative phase of the PDO is associated with anomalously cold sea surface temperatures (SSTs) in the tropical east and central Pacific, which enhance the zonal SST gradient across the equatorial Pacific. The latter drives a stronger Walker Circulation and a weaker Hadley Circulation, which leads to less convection and subsequently a warmer tropopause over the central equatorial Pacific. Over the North Pacific, positive sea level pressure anomalies occur, which damp vertical wave propagation into the stratosphere. This in turn slows the Brewer-Dobson circulation, and hence warms the tropical tropopause, enabling more water vapour to enter the stratosphere. The reverse chain of events holds for the positive phase of the PDO. Such ocean-troposphere-stratosphere interactions may provide an important feedback on the Earth’s global surface temperature.

The tropical tropopause layer (TTL) is the transition zone between the well-mixed, convectively active troposphere and the stably stratified and more quiescent stratosphere and hence a key region of troposphere-stratosphere coupling. The TTL acts as a “gate” for trace gases, and therefore significantly influences the composition of the entire upper atmosphere^1^. Water vapour is one of the most important stratospheric trace gases. The Air is frozen and dried to saturation, where the parcel encounters the coldest temperature. This occurs typically around the so called “cold point tropopause”, where the climatological vertical profile shows the coldest temperatures. Increasing stratospheric water vapour results in stratospheric cooling through infrared radiation (IR) emission, but tropospheric warming through IR absorption. It is through these radiative mechanisms that trends in stratospheric water vapour can impact global surface temperatures^2^,^3^. However, fundamental understanding of processes in the TTL, knowledge of long-term variability and trends of TPT, and links between TPT and tropospheric and stratospheric circulation patterns are still lacking^4^,^5^.

Various studies have reported, based on both radiosondes observations^6^,^7^,^8^ and reanalysis data^9^,^10^, a cooling of tropical TPT from the 1970s/1980s to the early 21^*st*^ century. This cooling has been linked to anthropogenic emissions of greenhouse gases (GHGs) which warm the troposphere and cool the stratosphere^11^. However, there are large uncertainties in TPT trends estimated from radiosonde data, because they do not provide global coverage and use different instruments and measurement practices^12^. Uncertainties exist as well in reanalysis data, which suffer from observation sparsity in the tropics and conflicts between different measurement types, e.g., a step-like discontinuity in some reanalysis data caused by the replacement from the Stratospheric Sounding Unit (SSU) to the Advanced Microwave Sounding Unit (AMSU) in 1998.

While anthropogenic GHG emissions have kept rising during the recent decades, the tropical TPTs did not decrease as expected from the GHG increase. Instead, since about the turn of the century, the tropical tropopause has significantly warmed, according to the Global Positioning System Radio Occultation (GPS-RO) measurements^13^. The GPS-RO measurements provide an unprecedented accurate, global, and weather-independent data set of tropopause temperature with high vertical resolution^5^,^14^. In fact, the tropical TPTs exhibit strong decadal to multidecadal variability^12^,^15^, which could be related to internal variability of the climate system^16^. At the same time, remarkable decadal variability has also been seen in the lower stratospheric water vapour^15^,^17^,^18^. So far, a consistent explanation of decadal to multidecadal variability in tropical TPTs as well as lower stratospheric water vapour is pending. Due to data limitations and application of a relatively simple theoretical framework, most of the aforementioned studies focused on zonal mean variability. Recent studies, however, have shown that the zonal structure of TPT variations is important, since it may reflect the mechanisms determining the TTL variability associated with climate change^19^,^20^. Investigating long-term variability with a focus on the zonal structure requires relatively long observations with high horizontal resolution, as well as suitable climate models.

In this study, we investigate the decadal variability of tropopause temperatures using the Modern Era Retrospective-Analysis for Research and Applications (MERRA) reanalysis for the time period 1979–2014, together with a series of extended-range (145 years) simulations with NCAR’s Community Earth System Model (CESM). As the GPS-RO data, the MERRA tropopause data resolve the main characteristics and long-term trends of TPTs, as shown below. The CESM, employing the Whole Atmosphere Community Climate Model (WACCM) as its atmospheric component with well resolved stratosphere (CESM-WACCM), was integrated with a fully-coupled ocean circulation model. A series of dedicated sensitivity experiments was conducted to detect potential mechanisms underlying decadal TPT variability.

## Results

### Interannual and decadal variability in TPTs and SSTs

**Figure 1:**
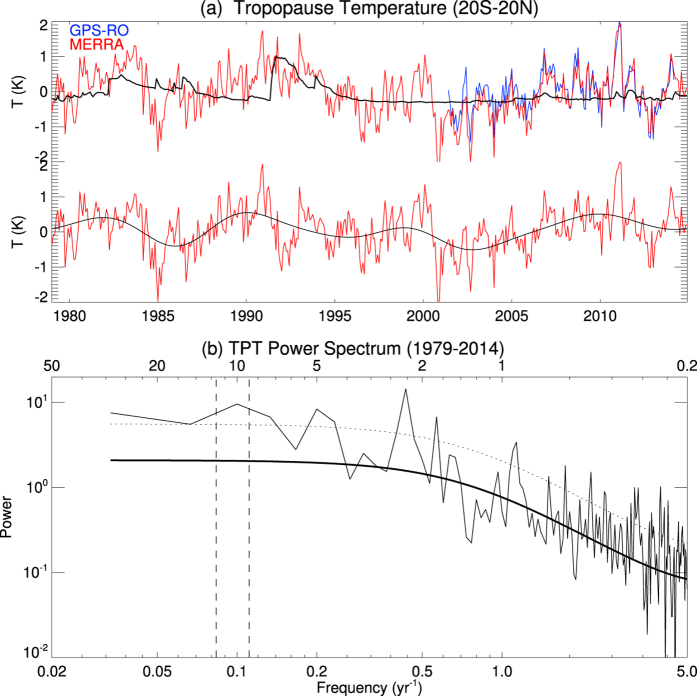
(**a**) Top Deseasonalized anomalies of tropical (20°S–20°N) tropopause temperatures from the GPS-RO (blue) and MERRA data (red). The black line indicates the influence of stratospheric aerosols estimated by linear regression (see details in “Data and Methods”). Bottom Tropical tropopause anomalies from MERRA data (red) where the influences of stratospheric aerosols has been removed according to the aerosol regression on top. The thick black line represents low-pass filtered (6 years) data. (**b**) Power spectrum of tropical tropopause temperatures from MERRA data without influences of stratospheric aerosol for the period 1979–2014. The thick black line indicates the best fit based on a first-order autoregressive model, whereas the dashed black line indicates the 95% confidence level. Dashed lines in the vertical indicate time periods of 9 and 13 years.

Figure 1a shows deseasonalized tropical TPT anomalies averaged over the region 20°S–20°N from both GPS-RO (2001–2014, blue line) and MERRA data (1979–2014, red line). Even though the MERRA data do not assimilate the GPS-RO data, the datasets are consistent with each other during the common period of 2001–2014. The MERRA data were shown to be well suited to investigate climatological behaviour^21^,^22^ as well as interannual variability. The black line shows the influence of stratospheric aerosols, as estimated by linear regression (see details in “Data and Methods”). The major volcanic eruptions of El Chichón (1982) and Mount Pinatubo (1991) warmed the tropopause obviously, while minor volcanic eruptions also influenced the TPTs, especially after 2001. This volcanic impact has been removed in the following figures in order to study the long-term behavior of tropical TPTs. The lower part of Fig. 1a also includes a low-pass filtered time series (retaining variability with periods of 6 years and longer, black line), and this indicates prominent decadal to multidecadal variability in tropical TPTs rather than a consistent long-term trend. The power spectrum of the MERRA TPT time series (Fig. 1b) depicts statistically significant peaks around 2, 5, and 10 years.

The 2-year peak is primarily related to the Quasi-Biennial Oscillation (QBO), which is the dominant mode of variability throughout the equatorial stratosphere, and also impacts TPTs^23^. The 5-year peak is connected to the El-Niño Southern Oscillation (ENSO) phenomenon^5^,^24^,^25^,^26^,^27^, which is the leading mode of global detrended monthly SST anomalies^28^.

**Figure 2:**
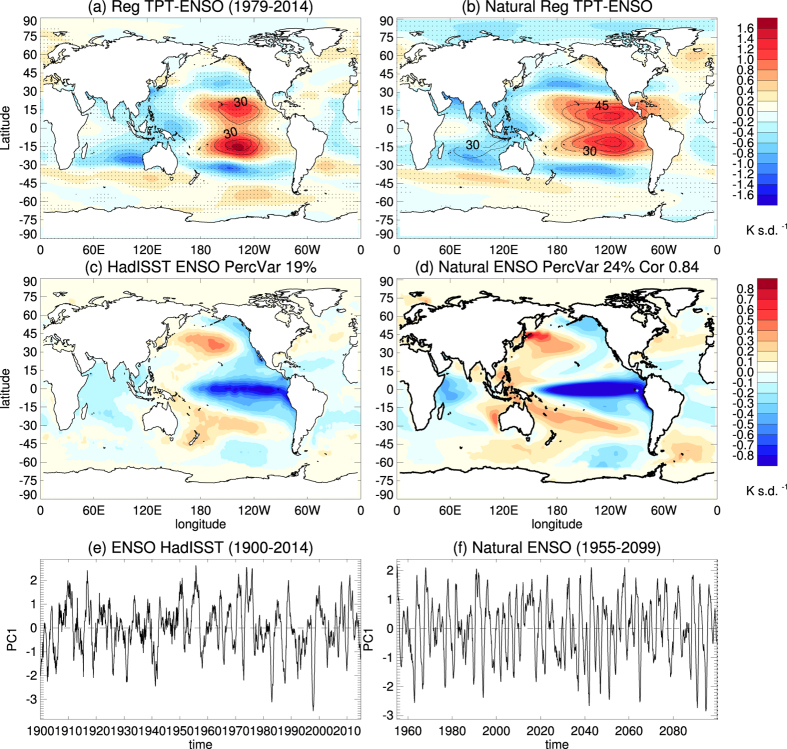
The regression pattern of (**a**) TPT from the MERRA data and (**c**) SST anomalies from the HadISST data on the ENSO index (**e**). The ENSO index is the leading PC of observed global SST anomalies from the HadISST data for the period 1900–2014, with the seasonal cycle and linear trend removed. The ENSO mode explains 19% of variance of the SST anomalies. Black contour lines in (**a**) show the explained variance in percent with a contour interval of 15%. Stippling in (**a**) indicates the 95% significance level, with autocorrelation effects taken into account. (**b**,**d**,**f**) Same as (**a**,**c**,**e**), but for the Natural CESM-WACCM experiment for the period 1955–2099. The right hand side number in (**d**) is the pattern correlation between (**d**,**c**). Maps were produced using licensed IDL (http://www.harrisgeospatial.com/ProductsandSolutions/GeospatialProducts/IDL.aspx), version 8.1.

The ENSO index, together with the associated SST anomalies and the responses in TPTs, are shown in Fig. 2. The ENSO index is the first principal component (PC) of global detrended SST anomalies, derived from the Hadley Centre SST analysis (HadISST)^29^. Note that the shown ENSO index and SST and TPT patterns correspond to a negative ENSO phase (La Niña), during which cold SST anomalies dominate the tropical central and eastern Pacific whereas warm SST anomalies occur in the western Pacific (Fig. 2c). The ENSO related TPT anomalies (TPT regressed onto the ENSO index) show positive anomalies over the tropical central Pacific, while negative anomalies dominate over the western Pacific and the Indian Ocean (Fig. 2a). This indicates a clear negative correlation between ENSO related SST anomalies and TPTs, which is consistent with previous studies^24^,^25^,^26^,^30^.

The 10-year peak indicates strong decadal variability in tropical TPTs and has been less considered in previous studies. Since the MERRA record is still relatively short hindering an accurate statistical assessment of TPTs on these timescales, long-term climate model simulations were analyzed for further insight into the potential drivers of decadal TPT variability, and to identify possible physical mechanisms.

The model was integrated in fully coupled mode for 145 years (1955–2099), i.e. with an interactive ocean and interactive atmospheric chemistry. A control run was done with natural external forcing (solar irradiance and volcanic aerosols) only, and additionally a nudged QBO^31^. GHG and ozone depleting substance (ODS) concentrations were kept constant at 1960 values throughout the integration. This experiment will be named “Natural” hereafter (see “Data and Methods” for details). We additionally carried out three simulations (FixSolar, FixSST and NOQBO) to detect the possible drivers and mechanisms of decadal TPT variability, in which each single driver (Solar, SST and QBO) has been set to constant values throughout the run (see details in “Data and Methods”).

**Figure 3:**
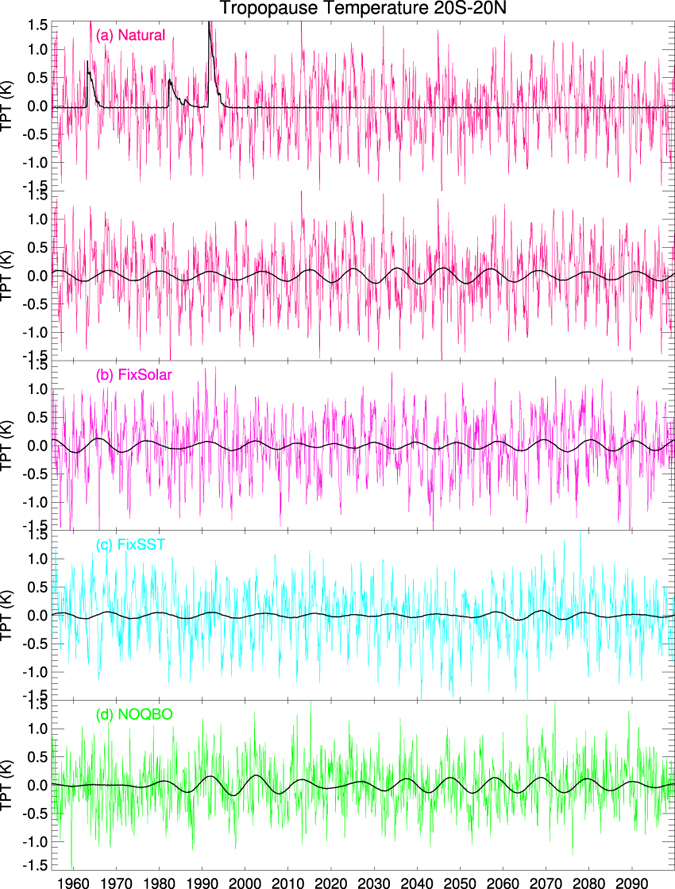
Deseasonalized anomalies of tropical (20°S–20°N) tropopause temperatures from different CESM-WACCM simulations: (**a**) the Natural run with volcanic aerosol forcing (black line in top figure) and without volcanic effects and 9–13 year band-pass filtered data (black line in bottom figure), (**b**) the FixSolar run, (**c**) the FixSST run and (**d**) the NOQBO run, with volcanic effects removed in (**b**–**d**), respectively.

TPT anomalies in the Natural experiment (Fig. 3a) show clear interannual fluctuations, and when the volcanic aerosol effect is removed, decadal variability becomes apparent. Comparing the 9–13 year band-pass filtered TPT time series of the Natural experiment, in which all drivers (i.e. interactive ocean, the 11-year solar cycle, and the QBO) are included, with the other model experiments, reveals interesting insights into the factors contributing to decadal to multidecadal variability (Fig. 3b–d). Switching off either the 11-year solar cycle or the interaction with the ocean reduces decadal TPT variability (Fig. 3b,c). Switching off the QBO nudging, but still allowing the solar cycle and the SSTs to vary, results in clear decadal tropical TPT variabilty (Fig. 3d). These model simulations suggest that both variable SSTs due to the interactive ocean and the 11-year solar cycle are important drivers of decadal variations in tropical TPTs.

**Figure 4:**
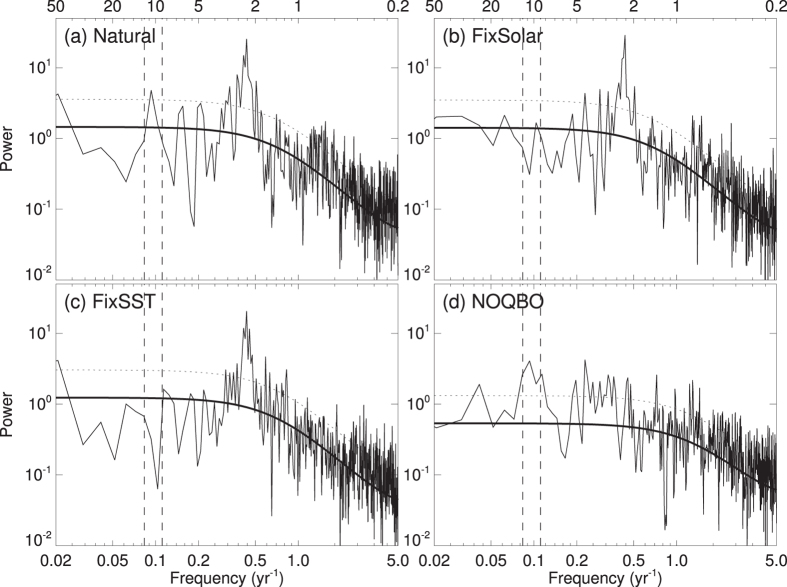
Power spectrum of tropical (20°S–20°N) tropopause temperatures from different CESM-WACCM simulations for the period 1955–2099: (**a**) the Natural run, (**b**) the FixSolar run, (**c**) the FixSST run and (**d**) the NOQBO run. Thick black lines indicate the best fit based on a first-order autoregressive model, and the dashed black lines indicate the 95% confidence level. The dashed vertical lines indicate time periods of 9 and 13 years. Influences from volcanic aerosols have been removed before applying the FFT analysis.

To compare the model’s behavior with the MERRA data shown above and to understand the importance of the individual forcing factors at interannual and decadal timescales, the TPT power spectra from the different model simulations are shown separately in Fig. 4. A clear and statistically significant peak at about 2 years can be found in the spectra of all model experiments except in the NOQBO experiment. This indicates that, with a nudged QBO, the model can simulate the QBO contribution to the 2-year peak seen in MERRA quite well (Fig. 1b). The 5-year peaks, which indicate the ENSO contribution to TPTs, are much weaker in the model simulations compared with MERRA (Fig. 1b). However, Fig. 2b,d show that the spatial ENSO pattern and the regressed TPT pattern in the Natural experiment is in very good agreement with the observed patterns (Fig. 2a,b). A significant decadal peak in the TPT power spectrum occurs in both the Natural and the NOQBO experiments (Fig. 4a,c), whereas the decadal peak is reduced in the FixSolar and the FixSST experiments (Fig. 4b,c), suggesting, as already shown in Fig. 3, that both the 11-year solar cycle, as well as varying SSTs due to the interactive ocean, contribute to decadal TPT variability.

### Relationship between decadal variability in SSTs and TPTs

One prominent source of climate variability on decadal to multidecadal timescales is the PDO, which is commonly defined as the leading mode of monthly SST anomalies over the North Pacific (20°–70°N)^28^. Though its definition is restricted to the North Pacific, it has global influences on the atmosphere, ocean, and marine ecosystems^32^. However, its impacts on the upper atmosphere, e.g. the stratosphere, have been less studied. Recent studies have examined the influence of the PDO and extratropical SSTs on the north polar stratosphere, and reported a weaker polar vortex and enhanced occurrence of stratospheric sudden warming (SSW) during positive PDO phases^33^,^34^,^35^.

**Figure 5:**
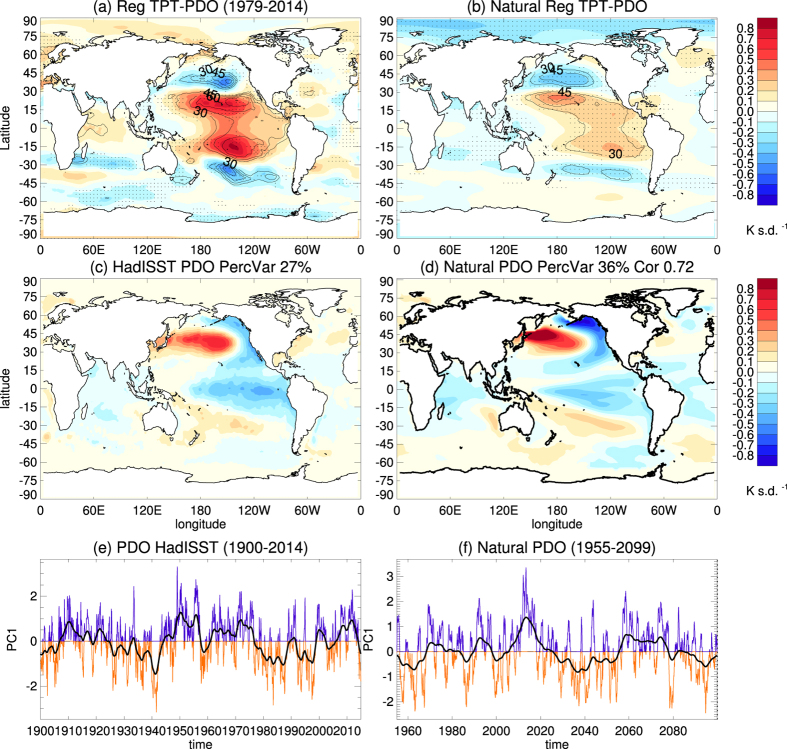
The regression pattern of (**a**) TPT from MERRA data and (**c**) SST anomalies from HadISST data regressed on the PDO index (**e**). The PDO index is the leading PC of observed SST anomalies over the North Pacific (20°–70°N, 110°E–100°W) from the HadISST data for the period 1900–2014, with the seasonal cycle and the global mean removed. The PDO mode explains 27% of the variance of the SST anomalies. The thick black line in (**e**) represents the low-pass (6-year) filtered PDO index. The time series of TPTs are first low-pass filtered and then regressed onto the filtered PDO index. TPT anomalies are derived from the MERRA data. The black contour lines in (**a**) show the explained variance in percent, whereas stippling in (**a**) indicates the 95% significance level, with autocorrelation effects taken into account. (**b**,**d**,**f**) Same as (**a**,**c**,**e**), but for the Natural CESM experiment from 1955–2099. The right hand side number in (**d**) is the pattern correlation between (**d**,**c**). Maps were produced using licensed IDL (http://www.harrisgeospatial.com/ProductsandSolutions/GeospatialProducts/IDL.aspx), version 8.1.

Figure 5 shows the PDO index, as well as the global regression patterns of SST and TPT anomalies from observations (Fig. 5a,c,e) and model simulations (Fig. 5b,d,f) onto the PDO index, which is the first PC of North Pacific SST anomalies. Note that the PDO index and the SST and TPT patterns correspond to a negative PDO phase. The observed PDO index shows pronounced decadal to multidecadal variations, which is especially apparent from its low-pass filtered time series (thick black line in Fig. 5e). A negative PDO phase is accompanied by cold SST anomalies in the tropical eastern and central Pacific and warm SST anomalies in the North Pacific^28^,^36^. This pattern of SST anomalies associated with the PDO (Fig. 5c) is similar to that associated with the ENSO (Fig. 2c) except that the PDO-related pattern has more pronounced signals in the North Pacific. These PDO associated SST anomalies have significant effects on TPTs, shown as warm anomalies over the tropical and subtropical east and central Pacific, and cold anomalies in the midlatitudes of both hemispheres (Fig. 5a). This explains up to 60% of TPT variance on the decadal timescale over the subtropical central Pacific. The PDO, therefore, has important effects on the decadal variability of the tropical TPTs.

A similar link between the PDO and TPT anomalies can be confirmed from the Natural experiment (Fig. 5b,d,f). The CESM-WACCM model reproduces the PDO induced SST pattern (Fig. 5d) very well compared to observations (Fig. 5c), with a pattern correlation up to 0.72. The response in TPTs from MERRA (Fig. 5a) is well simulated by CESM-WACCM (Fig. 5b), though the amplitude of TPT anomalies is slightly weaker than in MERRA. Both the HadISST and MERRA data and model results show a close relationship between PDO and TPT variability on the decadal timescale.

**Figure 6:**
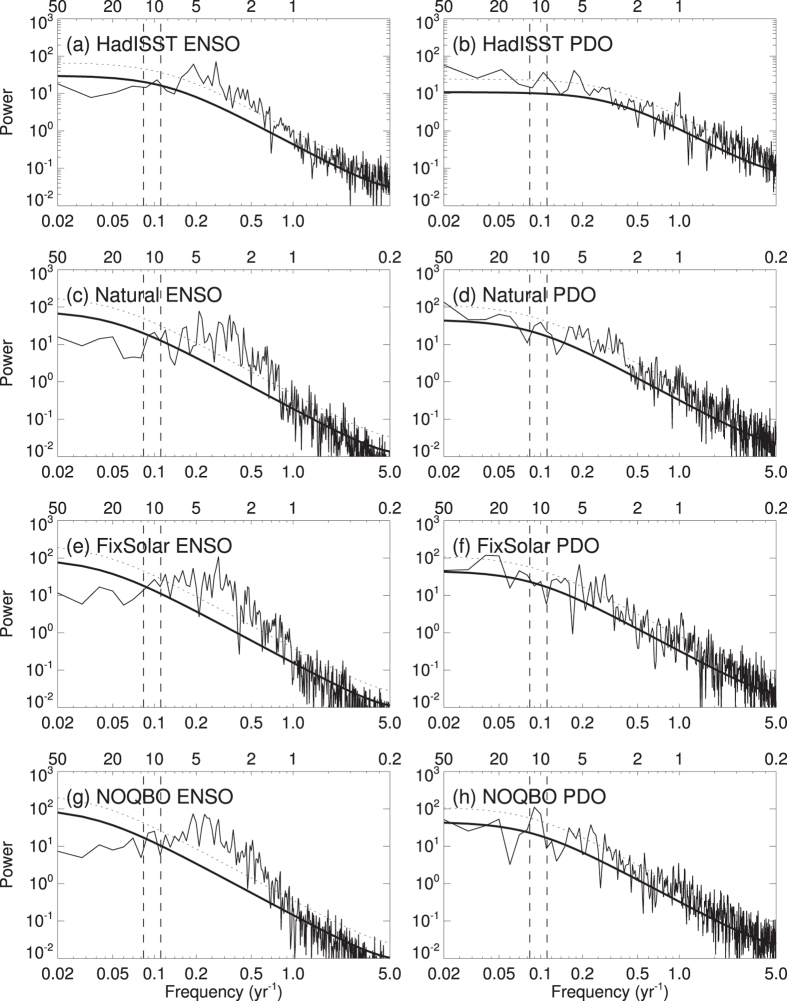
Power spectrum of observed (**a**) ENSO and (**b**) PDO indices from HadISST for the period 1900–2014. (**c**–**h**) Same as (**a**,**b**), but from the different CESM model experiments with coupled ocean, i.e. Natural, FixSolar, and NOQBO. The thick black lines indicate the best fit based on a first-order autoregressive model, whereas the dashed black lines indicate the 95% confidence level. The dashed vertical lines indicate time periods of 9 and 13 years. Influences from volcanic aerosols have been removed by a linear regression before applying the FFT analysis.

We now compare the power spectra of the ENSO and PDO indices (Fig. 6). From the model, only the experiments with an interactive ocean are shown as there is (by definition) no SST variability in the FixSST experiment. The power spectrum of the observed ENSO index (Fig. 2e) during 1900–2014 shows a strong peak around 3–6 years, which supports that ENSO dominates the 5-year peak in TPTs in Fig. 1b. All shown model experiments reproduce this statistically significant peak. The decadal peak in the spectrum of the observed PDO index (Fig. 5e) is statistically significant and suggests a PDO contribution to the decadal TPT variability. The power spectrum of the observed PDO index depicts another significant peak at about 5 years, which demonstrates the close connection between ENSO and PDO^37^. The PDO index from the Natural and the NOQBO experiments reproduce the observed decadal peak, although at the 90% significance level in the Natural experiment. The decadal peak in the FixSolar experiment disappears and suggests a potential synchronization of decadal SST variability with the 11-year solar cycle, similar to what has recently found in the North Atlantic^38^.

### Physical Mechanism for tropical TPT and SST connection

**Figure 7:**
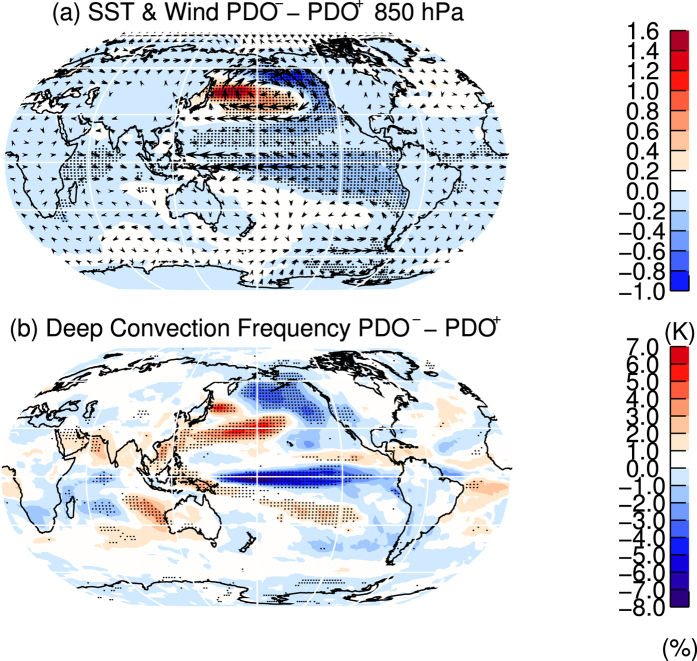
Composite differences between negative and positive PDO-phases from the Natural CESM experiment. Top Mean differences for SSTs (colour shading) and low-level (850 hPa) wind speeds (arrows). Bottom Mean differences in the deep convection frequency (colour shading). Stippling indicates the 95% statistic significance level, with autocorrelation effects taken into account. Maps were produced using licensed IDL (http://www.harrisgeospatial.com/ProductsandSolutions/GeospatialProducts/IDL.aspx), version 8.1.

To elucidate the mechanism that connects tropical TPTs and SSTs, we composited selected variables from the Natural experiment by positive and negative PDO-phases (shown as red and blue periods in Fig. 5f). During the negative PDO-phase (Fig. 7), low-level (850 hPa) easterly trade winds are enhanced, indicating a strengthening of the Walker Circulation over the equatorial Pacific, which is associated with anomalously cold equatorial Pacific SSTs, likely due to enhanced equatorial upwelling of water from subsurface levels. Anomalously warm SSTs and anticyclonic low-level circulation anomalies appear over the western North Pacific (Fig. 7a). The stronger Pacific Walker Circulation results in less frequent deep convection over the tropical central and western Pacific, and more frequent deep convection over the Maritime Continent (Fig. 7b). Deep convection lifts and cools the tropopause, and therefore a negative PDO phase contributes to a warmer tropopause in the tropical central and eastern Pacific, and a cooler tropopause over the Maritime Continent. This is seen in both the MERRA data and the model (see e.g. Fig. 5a,b).

**Figure 8:**
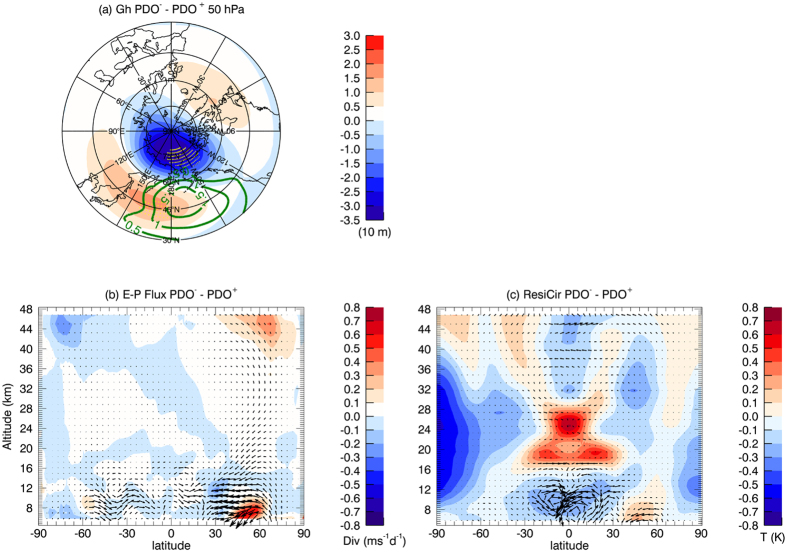
Composite differences between negative and positive PDO-phases from the Natural CESM experiment. Top Mean differences in sea level pressure (green contour lines) and geopotential heights in the middle stratosphere (50 hPa) on the northern hemisphere (colour shading) from 30°–90°N. Bottom Latitude-height sections for differences (left) in the E-P flux (arrows, indicating the direction of wave propagation) as well as its divergence (colour shading) and (right) in the residual mean meridional circulation (arrows, scaled with the square root of pressure, a direct diagnosis of meridional transport) and temperature differences (colour shading). The E-P flux and the residual mean meridional circulation are calculated from the Transformed Eulerian Mean (TEM) diagnostics^40^. Stippling indicates the 95% statistic significance level, with autocorrelation effects taken into account. Maps were produced using licensed IDL (http://www.harrisgeospatial.com/ProductsandSolutions/GeospatialProducts/IDL.aspx), version 8.1.

During the negative PDO-phase, there are positive sea level pressure anomalies over the North Pacific (Fig. 8a) as also suggested by the low-level winds (Fig. 7a), indicating a weakening of the Aleutian Low. The weaker Aleutian low generally interferes negatively with the average wave structure and weakens the climatological-mean stationary waves. Such negative interference damps the upward and poleward planetary wave propagation into the high-latitude extratropical stratosphere, which can be seen from the composite differences in the Eliassen-Palm flux (E-P flux) in Fig. 8b. This leads to a wave-induced strengthening of the polar vortex (Fig. 8a)^33^,^39^ and thus contributes to a weaker lower-stratosphere Brewer-Dobson circulation (Fig. 8c), according to the momentum budget^40^. This is consistent with recent studies which reported a weakening of the polar vortex during positive PDO phases^34^,^35^. The reduced vertical motion in the equatorial upper troposphere and lower stratosphere, which is a result from a combination of the weaker Hadley circulation in the upper tropical troposphere and the weaker Brewer-Dobson circulation in the tropical lower stratosphere, leads to a warming around the tropical tropopause at around 18 km, which is strongest near 15°N and 15°S (Fig. 7c). This mechanism is very similar to one previously reported with regard to the ENSO influences on the stratosphere^25^,^39^,^41^, but is applied here to the PDO and hence to the decadal timescale.

The results from the NOQBO experiment are in very good agreement with the results from the Natural experiment and indicate the robustness of our results (not shown). Even the FixSolar run shows a similar SST-TPT link, although the decadal peak in the power spectrum is rather small and shifted to multidecadal timescales (e.g., Figs 4 and 6).

**Figure 9:**
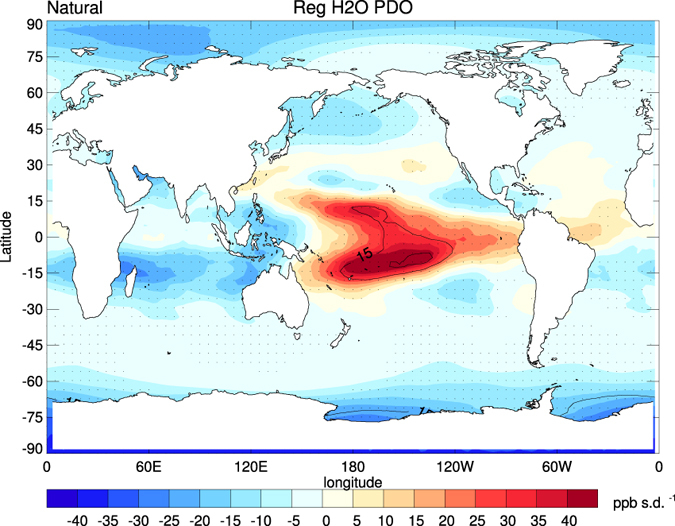
The regression pattern of lower stratospheric (85 hPa) water vapour anomalies on the low-pass (6-year) filtered PDO index (thick black lines in Fig. 5f) form the Natural CESM experiment. The time series of lower stratospheric water vapour are low-pass filtered before applying the regression. The black contour lines show the explained variance in percent. Stippling indicates the 95% significance level, with autocorrelation effects taken into account. Maps were produced using licensed IDL (http://www.harrisgeospatial.com/ProductsandSolutions/GeospatialProducts/IDL.aspx), version 8.1.

Figure 9 shows a similar regression of the PDO index than Fig. 5, but now for lower stratospheric (85 hPa) water vapour anomalies from the Natural run. The regression pattern in lower stratospheric water vapour anomalies shows positive anomalies over the tropical Pacific and Atlantic, and negative anomalies over the Indian Ocean. This suggests a negative correlation between SSTs and lower stratospheric water vapour anomalies and is consistent with the previously shown negative correlation between SSTs and TPTs (Fig. 5). The warmer the tropopause, the more water vapour can enter the stratosphere. This can explain about 15–30% of the water vapour variance on decadal timescales.

## Summary and Discussion

The tropical tropopause temperature (TPT), as shown by the MERRA reanalysis data, exhibits clear decadal variability between 1979 and 2014. This decadal variability in tropical TPTs may be related to the Pacific Decadal Oscillation (PDO), the leading mode of monthly SST anomalies over the North Pacific. This has been shown by the MERRA TPTs and observed SSTs (HadISST) for the real climate behaviour, and also by means of a number of long-term climate model simulations with the CESM-WACCM model.

We suggest that tropical TPTs, which control the amount of stratospheric water vapour, could be in part forced by decadal to multidecadal variability in the equatorial Pacific and the North Pacific sea surface temperatures (SSTs). A PDO associated SST pattern modulates tropical TPTs via changes in the Walker, the Hadley, and the stratospheric Brewer-Dobson circulation. We present evidence for a PDO modulation of TPTs and lower stratospheric water vapour in the Pacific sector similar to what has been proposed for the Atlantic where the Atlantic Multidecadal Oscillation/Variability (AMO/V) may impact multidecadal variability in the strength of the stratospheric polar vortex^42^. Whether and how these stratospheric changes feed back on the ocean awaits further studies.

The model experiments also suggest that decadal variability in TPTs might be modulated by the 11-year solar cycle, although the currently available observational record is still too short to investigate this. Further investigations with longer observational records and other coupled-climate models are needed to confirm the results shown by the CESM-WACCM model, and to give a comprehensive understanding of the mechanisms in determining decadal variability of tropical tropopause temperatures.

## Data and Methods

### The MERRA reanalysis Data

The Modern Era Retrospective-Analysis for Research and Applications (MERRA) is a reanalysis created by NASA’s GEOS-5 data assimilation system, which includes many modern observing systems (such as EOS) in a climate model framework^21^. The native grid has 72 vertical levels up to 0.01 hPa through the stratosphere, about 1 km resolution in the UTLS, and a horizontal resolution of around 1/2° × 2/3° (latitude × longitude). Tropopause data from MERRA were downloaded from the single-level diagnostic products at native horizontal resolution.

Even though reanalysis data are a combination of observational data with a numerical model, they provide a consistent and best estimate of the real atmospheric temperature and circulation. As one of the most advanced reanalysis, MERRA tropopause temperature shows the best climatological characteristics as radiosonde observations, compared with other reanalysis^22^. MERRA is also suited to study the long-term variability in tropical tropopause temperatures, since the discontinuity caused by the change from the SSU to the AMSU as mentioned in the Introduction is relatively small^21^.

### Model and Simulations

NCAR’s CESM model (version 1.0.2) is a fully coupled climate model, including an interactive ocean (POP2), land (CLM4), sea ice (CICE) and atmosphere (CAM/WACCM) components. The atmospheric component, WACCM (version 4) is a chemistry–climate model, with detailed middle atmospheric chemistry and dynamics, extending from the surface to about 140 km^43^. The standard version has 66 vertical levels, which means about 1 km vertical resolution in the TTL. All simulations use a horizontal resolution of 1.9° × 2.5° (latitude × longitude) for the atmosphere and approximately 1 degree for the ocean.

The Natural run covers the period from 1955 to 2099. It employs all natural forcing agents including spectrally resolved solar variability, a fully interactive ocean, volcanic aerosols and a nudged QBO^31^. The solar forcing is based on observations 1955–2004, while the future projection (2004–2099) is a repetition of the last four observed 11-year solar cycles. Volcanic aerosols follow the SPARC (Stratospheric Processes and their Role in Climate) CCMVal (Chemistry-Climate Model Validation Activity) REF-B2 scenario recommendations^44^. In brief, volcanic aerosol surface area density (SAD) is prescribed from a monthly zonal-mean time series derived from observations 1955–2004 and background values without major volcanic eruptions 2005–2099. The QBO forcing time series is determined from the observed climatology of 1953–2004 via filtered spectral decomposition of that climatology. This gives a set of Fourier coefficients that can be expanded for any day and year in the past and the future. Anthropogenic forcing like GHGs and ODSs are set to constant 1960s conditions. Besides the Natural run, a number of different CESM-WACCM simulations were used for comparison and to disentangle physical mechanisms, by switching out individual forcing factors, i.e., the solar cycle (FixSolar experiment, without solar cycle variability), the QBO (NOQBO experiment, without QBO nudging), and the SSTs (FixSST experiment, prescribed SST as climatological monthly varying values from the Natural run). The TPT as defined by the WMO^45^ is a direct output of the model.

### Removing Volcanic Influences

The observed aerosol data is used to estimate the stratospheric aerosol influences on tropopause temperatures as shown in Fig. 1. It is based on observed stratospheric aerosol optical depth (AOD) and has been constructed for the CCMI project (ftp://iacftp.ethz.ch/pub_read/luo/ccmi/). The linear regression has been applied separately for major and minor volcanic eruptions since the AOD values are much higher in the former than in the later: the first step uses the whole time series of AOD 1979–2014 in the regression, indicating the major volcanic effects; the second step uses the AOD time series within one standard deviation only, to estimate the influences from minor volcanic eruptions. For model simulations (Fig. 3), the volcanic influences are estimated using volcanic aerosol data described above in “Model and Simulations”, which includes major volcanic eruptions only.

## Additional Information

**How to cite this article**: Wang, W. *et al*. Decadal variability of tropical tropopause temperature and its relationship to the Pacific Decadal Oscillation. *Sci. Rep.* **6**, 29537; doi: 10.1038/srep29537 (2016).
